# The cholera outbreak in Yemen: lessons learned and way forward

**DOI:** 10.1186/s12889-018-6227-6

**Published:** 2018-12-04

**Authors:** Frederik Federspiel, Mohammad Ali

**Affiliations:** 1 Department of Global Health and Development, London School of Hygiene & Tropical Medicine, 9 Tavistock Place, London, WC1H 9SH UK; 2 Department of International Health, Johns Hopkins Bloomberg School of Public Health, 615 N. Wolfe Street, Baltimore, MD 21205 USA

**Keywords:** Cholera, Yemen, Conflict, Humanitarian response, Oral cholera vaccination, WaSH

## Abstract

The Yemen cholera outbreak has been driven by years of conflict and has now become the largest in epidemiologically recorded history with more than 1.2 million cases since the beginning of the outbreak in April, 2017. In this report we review and discuss the cholera management strategies applied by the major international humanitarian health organizations present in Yemen. We find the response by the organizations examined to have been more focused on case management than on outbreak prevention. Oral Cholera Vaccines (OCVs) were not delivered until nearly 16 months into the outbreak. A recent scale-up of the global OCV stockpile will hopefully allow for rapid mass deployment of the OCV in future humanitarian emergencies. Continuous funding to this stockpile will be crucial to maintain this option for prevention and control of cholera outbreaks. Of equal importance will be the timely recognition of the need for mass OCV deployment and development of more specific, comprehensive and actionable evidence-based frameworks to help guide this decision, however difficult this may be. The outbreak highlights the importance for international humanitarian health organizations to have a continuous discussion about whether and to what extent they should increase their focus on pre-emptively addressing the environmental determinants of communicable diseases in humanitarian emergencies. Strong advocacy from the public health community for peace and the protection of human health, by bringing to attention the public health impacts of armed conflict and keeping the world’s political leaders accountable to their actions, will remain crucial.

## Background

The United Nations have deemed the situation in Yemen the world’s worst humanitarian crisis [1]. In the midst of this, the largest and fastest spreading cholera outbreak recorded since the WHO began recording cholera outbreaks in 1949 has erupted [2, 3]. As of October 7, 2018, the WHO estimates a cumulative total of 1,236,028 reported cases of cholera to have occurred in Yemen since the beginning of the outbreak in April, 2017 [4]. 2556 of these cases have been fatal, yielding a case fatality rate of 0.21% [4]. Children constitute 58% of reported cases [5].

How could an outbreak of this scale occur? Already before the conflict erupted, approximately half of all children under 5 were stunted reflecting a high prevalence of chronic malnutrition, lowering their resilience towards cholera [6, 7]. Water, Sanitation and Hygiene (WaSH) infrastructure was limited [8]. Destruction of the fragile WaSH bulwark against water-borne disease started when bombings commenced in March, 2015 [9]. Over the past 3 years, key infrastructure in Yemen has been destroyed, hampering the possibility of prevention and control of cholera in the country. The main seaport to Yemen, Al-Hudeydah, has been bombed and later blocked, disrupting the supply of aid and other supplies into the country [10, 11]. The bridge that used to serve as the main supply route to the capital, Sana’a, has been bombed and so have hospitals and other medical facilities [3, 9, 12–14]. The United Nations Office for the Coordination of Humanitarian Affairs (UNOCHA) have estimated that “*16 million* [people] *lack access to safe water and sanitation, and 16.4 million lack access to adequate healthcare*” [15]. The costs of food, water, fuel, medicines and other basic commodities have increased dramatically [16–18], lowering overall resilience towards cholera in Yemen even further.

It is clear that such a collapse of infrastructure has created fertile ground for a cholera outbreak, and that the critical triggering cause was conflict [9, 15, 17, 19–22]. Both this cause of the problem and its ultimate solution lie in the political realm, and we strongly advocate for immediate political resolution with involvement from a so far neglectful international community [23–25]. However, cholera outbreaks frequently occur on the backdrop of conflict and/or poor WaSH infrastructure yet fail to spread so rapidly or to reach this magnitude [26–30]. In this debate piece we wish to examine if there are any lessons for the humanitarian and public health community that can be taken away from the humanitarian response in Yemen to better equip us to do our part in preventing future outbreaks from reaching this scale.

We reviewed publicly available information on the official websites and in reports from the major international health organizations involved in the humanitarian response in Yemen in December 2017, and academic English language literature on the Medline, Embase and Global Health databases published from 2015 and onward until submission in May 2018 (search string: “Yemen AND cholera”), to inform a discussion of potential shortcomings and lessons to be learned from the humanitarian response by health organizations. Our academic literature search yielded 40 results, the relevant of which are included in this paper. Our review findings are summarized in Table 1.

**Table 1:** Summary of some of the key activities of the major international humanitarian health organizations in Yemen as stated on their official websites in December, 2017, and in the academic literature reviewed by May, 2018. This is not an exhaustive list, but may serve as an indicator of some of the main focus areas of these individual organizations during the conflict up until the reviews were conducted. (Some categories may overlap)

Key activities	WHO [22, 31, 33, 34]	UNICEF [35, 36]	IRC [37, 38]	ICRC [22, 39]	MSF [40, 41]
Delivery of new health services	Operated 406 general health and nutrition mobile teams		Delivered health and nutrition services, essential drugs and medical supplies		Established 37 cholera treatment centers and oral rehydration points
	Delivered child and nutrition interventions in 323 districts		Deployed mobile health teams		
	Established 36 cholera treatment centers		Established community health volunteer networks for referral of malnourished children to treatment and counsel		
	Established 139 oral rehydration corners				
Support of existing health system	Sustained functionality of 414 health facilities		*See “Health infrastructure”*	Supported 17 cholera treatment facilities	
Health infrastructure	Distributed more than 2 million liters of fuel for hospital generators, ambulances etc.	Delivered 40 tons of medical equipment including medicine, ORS, IV fluids and diarrhea kits.	Provided seven hospitals with drugs, supplies and infrastructure	Sent medical supplies including IV fluids, ORS, antibiotics and chlorine tablets for cholera management	
	Delivered 1 million bags of IV fluids				
	Distributed 158 cholera kits				
	Sent 1450 cholera cots				
	Shipped in ambulances				
WaSH		Provided safe water to over one million people	Delivered WaSH services	Dispatched engineers to restore water supply systems in the country	Planned to conduct outreach activities including chlorination of infected water sources and distribution of hygiene kits
		Deployed 20,000 community hygiene promoters (part of awareness campaigns below)			
Staff training	Trained 900 health workers in cholera management		Trained health staff on cholera treatment		
Epidemiological activities	Expanded disease surveillance capacity				
Management	Coordination of Health Cluster Yemen	WaSH cluster lead			
Awareness		Ran several cholera awareness campaigns			Planned to conduct awareness-raising via radio stations and mosques
Advocacy			Advocated for a direct humanitarian air service and for peace		
Summary output of activities	Treated 700,000 suspected cases of cholera			Provided care to nearly one in five cholera cases in Yemen	Admitted more than 103,000 patients

*Abbreviations*: *WHO* World Health Organization, *UNICEF* United Nations Children’s Fund, *IRC* International Rescue Committee, *ICRC* International Committee of the Red Cross, *MSF* Médecins Sans Frontières, *WaSH* Water, Sanitation and Hygiene, *ORS* Oral Rehydration Solution

## Main text

### International humanitarian health organization responses and funding shortfalls

The Yemen Health Cluster consists of 40 member organizations and is coordinated by the WHO [31]. In June, 2017, the WHO and the Health and Water Sanitation and Hygiene (WaSH) Clusters in Yemen published a response plan for the cholera outbreak [32]. The plan included two principal approaches to cholera management: a) treating all cases of Acute Watery Diarrhea (AWD) as cholera without the need for lab confirmation in areas where cholera has been previously culture confirmed, and b) early response epidemiological and case management activities when a suspected case is reported in an outbreak-free area. These actors also reported planning to explore the feasibility of an Oral Cholera Vaccination (OCV) campaign [32].

As part of their general response in Yemen, the WHO described having sustained functionality of 414 health facilities, operation of 406 general health and nutrition mobile teams and delivery of child and nutrition interventions in 323 districts, having established 36 cholera treatment centers, expanded disease surveillance capacity and having distributed more than 2 million liters of fuel for hospital generators, ambulances etc. [33]. Specifically, the WHO reported having established 139 oral rehydration corners, trained 900 health workers in the management of cholera, delivered 1 million bags of IV fluids, distributed 158 cholera kits and sent 1450 cholera cots [34], and they had shipped in ambulances as well [22]. Through these and other interventions the WHO described having treated 700,000 suspected cases of cholera [34].

UNICEF described having provided safe water to over one million people and delivered 40 tons of medical equipment including medicine, Oral Rehydration Solution (ORS), IV fluids and diarrhea kits [35]. UNICEF serves as the WaSH cluster lead and reported having run several cholera awareness campaigns including the deployment of 20,000 community hygiene promoters [35, 36].

On their webpage for Yemen, the International Rescue Committee (IRC) described delivering health, nutrition, WaSH services, essential drugs and medical supplies, training health staff on cholera treatment and advocating for a direct humanitarian air service and for peace [37]. In a *Lancet* appeal, IRC staff further described providing seven hospitals with drugs, supplies and infrastructure, deploying mobile health teams and establishing community health volunteer networks for referral of malnourished children to treatment and counsel [38].

The International Committee of the Red Cross (ICRC) reported having sent medical supplies including IV fluids, ORS, antibiotics and chlorine tablets for cholera management, supported 17 cholera treatment facilities and provided care to nearly one in five cholera cases in Yemen, and they had dispatched engineers to restore water supply systems in the country [22, 39].

Médecins Sans Frontières (MSF) described having admitted more than 103,000 patients to 37 cholera treatment centers and oral rehydration points [40] and planning to conduct outreach activities including chlorination of infected water sources, distribution of hygiene kits and awareness-raising via radio stations and mosques [41].

While the above is not an exhaustive list of all the activities of these organizations, it should at least provide an overview of some of their main focus areas in Yemen up until the time of review. It is also important to note that it was not within the scope of this paper to review the activities of all major engineering and infrastructure organizations present in Yemen, as well as the examined health organizations, and the following discussion of sanitation efforts will pertain only to organizations with a health mandate. Furthermore, the humanitarian crisis in Yemen has been neglected by the international community [23–25], and any criticism of the humanitarian response presented in this paper should reflect back to other stakeholders such as donors, politicians and other decision makers that have failed to allocate sufficient resources to this crisis. In regards hereto, the IRC has pointed to the fact that “only 47% of the UN’s 2016 response plan was funded, leaving a shortfall of US$868 million to cover the basic needs of 14 million people” [38], while the WHO experienced a funding gap of more than 80% in late 2015 for their activities in the Yemen health cluster [42, 43]. At the High-Level Pledging Event for the Humanitarian Crisis in Yemen on April 25, 2017 in Geneva, US$1.1 billion was raised, falling short US$1 billion from the UN estimate of what would be needed “*to prevent a full-blown humanitarian catastrophe in Yemen*” [22]. An additional funding appeal at the event for $250 million specifically for combatting cholera yielded only $47 million [22]. This was eventually followed up by a US$2 billion funding pledge at a new High-Level Pledging Event in April, 2018, with financial contributions among others coming from Saudi Arabia, the United Arab Emirates and Kuwait, which are member countries of the Saudi-led coalition, and the United States, the United Kingdom and France, which are supporting countries [44–50].

### Oral cholera vaccines

A striking observation from our review of the humanitarian response is the absence of delivering OCVs in any of the examined organizations’ attempts to control the outbreak, except for the WHO, Health and WaSH clusters’ description of plans to explore the feasibility of OCVs. At the time (December, 2017), nearly 1 million cases had already occurred [51]. In July 2017, 3 months into the outbreak, 500,000 OCVs had been scheduled to arrive in Sana’a, but they were redirected because the request was cancelled by the requestor [52]. A concern raised by some aid agencies was that a mass immunization would be logistically difficult and that the impact of vaccination would be minimal due to the scale of the outbreak [53]. In a *Lancet Gastroenterology & Hepatology* editorial it is stated that “… *plans for a vaccination effort were deemed futile and scrapped, citing security issues, challenges in distribution and administration, and the sheer scale of the epidemic...*” [22]. In August 2018, nearly 16 months into the outbreak, OCVs were finally delivered to 540,000 people by the WHO and UNICEF, and a follow-up round was conducted in September reaching about 387,000 of these people [54, 55].

OCVs for emergency settings are stored in a donor-funded stockpile managed by the International Coordinating Group on Vaccine Provision (ICG) [5]. A recent meta-analysis of seven randomized trials and six observational studies estimates the mean effectiveness of standard two-dose killed oral cholera vaccination at 76% and mean efficacy at 58%, with protection lasting for at least 3 years [56]. Oral cholera vaccination has been demonstrated to be safe, logistically feasible and acceptable by the recipient population [56–60], as well as inexpensive in various settings, with total costs including procurement and delivery per fully vaccinated individual of less than US$10 [61–65].

Accepting the unacceptable premise that the Yemeni conflict became reality, the question that arises is: Could the largest cholera outbreak ever recorded have been avoided or at least managed, had enough OCVs been deployed earlier on in the conflict?

The solely theoretically based answer to this question is: Most likely, given the available evidence of the qualities of OCVs. However, considering the nature of this humanitarian emergency, this answer becomes little more than an *ex post facto* rationalization of a deeply chaotic situation that, as indicated above, most probably did not lend itself to orderly and comprehensive public health interventions such as mass vaccination.

Human Rights Watch have reported utterly chaotic conditions in Yemen [66]. If their reports are anywhere close to the truth, then that would provide an explanation of why mass OCV campaigns may simply not have been possible earlier on in the conflict, as indicated by the *Lancet Gastroenterology & Hepatology* editorial mentioned earlier [22]. Also, given the fact that more than two million Yemenis are internally displaced within their country [67], and that many likely are internally trapped, mass public health interventions are nowhere as easily carried out as in a refugee camp setting in a country not immersed in conflict.

However, this explanation is challenged by the magnitude of the other humanitarian operations that it evidently has been possible to carry out in Yemen. In light hereof, some level of criticism may be warranted of the international humanitarian response as being very adept at managing cases when the outbreak had begun, as reflected by the impressively low case fatality rate of 0.21%, but ill-equipped in terms of preventing a cholera outbreak in the approximately 2 years of conflict and humanitarian presence that preceded the outbreak (this view is shared by Dr. Anita Zaidi in a recent opinion piece in *Nature* [68]), or controlling it once it had occurred.

### Moving forward

Concerning water and hygiene interventions, it appears that the Health and WaSH clusters, the IRC, MSF and ICRC have been thoroughly engaged. However, we were unable to document any major dedicated sanitation interventions carried out by the international humanitarian health organizations investigated. As mentioned, this may likely be due to the type of organizations examined, but nevertheless it leads to the question of whether and to what extent international humanitarian health organizations should increase their focus on addressing the environmental determinants of health rather than managing the cases they generate. This is a core discussion in humanitarian health that it is not within our mandate nor our abilities to answer, but the case of Yemen underlines the necessity of having this discussion on a continuous basis within each implementing humanitarian health organization. Going forward, given the current breakdown of sanitation systems [15] and the key importance of sanitation for prevention of a cholera outbreak in areas where the population has not been exposed and has no immunity to cholera, as well as for prevention of other water-borne disease outbreaks, sanitation has to be a key priority in the ongoing humanitarian response and the future development work when the conflict settles.

OCVs were not delivered until nearly 3.5 years into this humanitarian emergency, which has most likely been due to ongoing conflict, logistical circumstances, the scale of the epidemic, impairment of the humanitarian response by the parts to the conflict and some degree of negligence from donors, politicians and other decision makers. Whatever the reasons, OCVs were not distributed until nearly 16 months into the cholera outbreak by which time more than a million cases had accumulated. Neither were they in the two years of WaSH infrastructure breakdown that preceded the outbreak. This should serve as a historic example of the failure to control the spread of cholera given the tools that are available. Today, “*cholera outbreaks are entirely containable*” (*The Lancet* editorial, 2017) [6].

Based on their experience with the cholera outbreak in Juba, South Sudan, Parker et al. (2017) advocate among other things for improved laboratory and surveillance capacity in countries to be able to declare outbreaks faster, and for simplification of the mechanisms to access the ICG stockpile to allow for timely deployment of vaccines [69].

Single-dose killed oral cholera vaccination has been attempted, and the strategy has shown a mean short-term effectiveness of 69% in meta-analysis based on two studies [56], and 40% efficacy based on one trial [70]. This could serve as a potential option for when logistical and security constraints render the standard regimen impossible [56, 60, 69–71]. The strategy however remains contended [72, 73] and needs further evaluation. Live-attenuated OCVs have been developed and shown promising results for single-dose administration [74], however their cost is likely going to be a significant barrier to their use in mass vaccination campaigns.

The 2011 Sphere Handbook applicable in the Yemen conflict did not specifically recommend reactive or pre-emptive mass OCVs [75], but it was also written before the advent of the ICG OCV stockpile. The available draft of the upcoming 2018 version includes this, and we warmly welcome this progress [76]. The ICG stockpile has now expanded from 2 million doses entering the stockpile per year in 2013 to over 25 million doses expected annually from 2018 and onwards [77]. This scale-up will be a crucial improvement of the ability to control cholera in emergency settings, and sustained funding to maintain this stockpile will be essential for controlling future outbreaks.

Assessment of the need for mass OCVs should happen pre-emptively in a humanitarian emergency. Regarding this issue, Qadri et al. (2017) and Parker et al. (2017) argue for the development of validated predictive tools to help guide the decision on when and to which humanitarian emergencies OCVs should be deployed, a notion we do support, whilst acknowledging the difficulty of the task of predicting the outbreak of cholera [53, 69]. The current WHO framework for decision-making on vaccination in acute humanitarian emergencies offers a tool for assessing the risk level for a cholera outbreak, and in the case of Yemen, the high-risk criteria were fulfilled [78]. The current WHO position paper on cholera vaccines offers general advice in four sentences on which factors to consider regarding the use of OCVs for cholera control in humanitarian emergencies [79], while the available draft of the 2018 Sphere Handbook offers further general advice on this [76]. In the end, the decision has to be made on a case-by-case basis by the people involved in a given humanitarian response, leaving room for a degree of arbitrary in the decision-making process that should at least be sought to be minimized through the development of more specific, comprehensive and actionable frameworks based on the accumulating and promising evidence from mass OCV campaigns in humanitarian emergencies [26, 29, 60, 71, 80–84].

Based on the example of Yemen, Dureab et al. (2017) argue for the institution of emergency preparedness systems by countries in conflict capable of preventing epidemics [21]. While we do applaud this thought as viewed in isolation, we see considerable political, institutional and logistical barriers to such systems during active conflict, and believe that part of this mandate therefore necessarily has to remain in the hands of international impartial institutions such as the UN organizations.

Finally, in terms of advocacy, *The Lancet* editorial board notes that high-level attention for Yemen is necessary with continued advocacy including at the UN level for the protection of health in conflict situations [25]. We will add that public health professionals and -academics are in a strong position to join in by applying their knowledge and skills in advocacy efforts for peace and the preservation of public health in conflict and to keep the world’s leaders accountable for the health and human rights consequences of their actions.

## Conclusions

The conflict in Yemen has caused the largest cholera outbreak in epidemiologically recorded history. OCVs were not delivered pre-emptively, nor until nearly 16 months into the outbreak, during which time more than a million cases occurred. We are, hopefully, entering into a new era in which OCVs will be amply available for rapid disbursement to humanitarian emergencies. Continuous funding to the ICG OCV stockpile will be crucial to maintain this option for prevention and control of cholera outbreaks. Of equal importance will be the timely recognition of the need for mass OCV deployment and development of more specific, comprehensive and actionable evidence-based frameworks to help guide this decision, however difficult this may be. The Yemen cholera outbreak highlights the importance for international humanitarian health organizations to have a continuous discussion about whether and to what extent they should increase their focus on pre-emptively addressing the environmental determinants of communicable diseases in humanitarian emergencies. Finally, strong advocacy from the public health community for peace and the protection of human health, by bringing to attention the public health impacts of armed conflict and keeping the world’s political leaders accountable to their actions, will remain crucial.

## Abbreviations

AWD: Acute Watery Diarrhea
ICG: International Coordinating Group on Vaccine Provision
ICRC: International Committee of the Red Cross
IRC: International Rescue Committee
MSF: Médecins Sans Frontières
OCVs: Oral Cholera Vaccines
ORS: Oral Rehydration Solution
UNICEF: United Nations Children’s Fund
UNOCHA: United Nations Office for the Coordination of Humanitarian Affairs
WaSH: Water, Sanitation and Hygiene
WFP: World Food Programme
WHO: World Health Organization
